# The ethical foundations of patient-centered care in aesthetic medicine

**DOI:** 10.1186/s13010-024-00151-1

**Published:** 2024-02-05

**Authors:** Editta Buttura da Prato, Hugues Cartier, Andrea Margara, Beatriz Molina, Antonello Tateo, Franco Grimolizzi, Antonio Gioacchino Spagnolo

**Affiliations:** 1 Private Practice, Verona, Italy; 2 Centre Medical Saint-Jean, Arras, France; 3 Plastic Surgery Service, Turin, Italy; 4 Medikas Clinic, Somerset, UK; 5 Private Practice, Pavia, Italy; 6 Private Practice, Milan, Italy; 7 IBSA Farmaceutici Italia, Lodi, Italy; 8 Institute of Bioethics and Medical Humanities, Faculty Member, Catholic University of the Sacred Heart, Rome, Italy

**Keywords:** Aesthetic medicine, Ethics, Patient-centricity, Care

## Abstract

This article addresses some critical aspects of the relationship between aesthetic medicine (AM) and ethics and proposes a possible deontological ethical line to pursue based on current practices. The role of AM has always been controversial and suffers from unclear practical and moral boundaries, even within academic settings, since it aims to improve the appearance of individuals, not to cure a disease. Today, it is essential and pertinent to discuss these issues, as AM specialists are dealing with a growing and increasingly demanding patient population that has undergone profound evolution in recent years. Current challenges within the field of AM include a lack of global uniformity concerning the education of AM specialists, an increasing number of physicians practicing AM with diverse training backgrounds, the spread of AM being practiced outside of medical practice or hospital settings, and the influence of social media where the success is modelled and dictated by the identification of a youthful appearance). By the field of action enriched by technologies that aim not only at enhancement per se but also at the preservation and regeneration of tissues, it is necessary to establish an active multidisciplinary discussion on the definition of shared ethical limits. This discussion would allow AM to fully reclaim its identity as a specialty that aims to improve patient well-being whilst maintaining respect for patient aesthetic harmony, the expertise of specialists who practice AM, the essential role of safety, and awareness of the importance of a confidential doctor−patient relationship.

## Introduction

### Words matter

Aesthetic medicine (AM) is an umbrella term currently used for describing specialties with the aim of improving physical appearance through invasive and non-invasive procedures (mostly elective) to treat aesthetic alterations and unaesthetic sequel of illnesses or injuries and prevent aging for patient satisfaction [1]. Procedures to enhance body appearance date back to ancient civilizations and have changed according to culture and aesthetic standards; however, AM practices have generally always been regarded as separate from specialties focused on healing and healthcare [2]. Investigating the etymological meaning of AM, we discover that the word “medicine” comes from the Latin *medeor* (i.e., to mend), whilst “aesthetics” is derived from the ancient Greek *aìsthesis*which means knowledge through the senses, the perception of everything that can be experienced, the beautiful as well as the ugly [3].

Ethics is an idea embedded in the concept of medicine, transcending any procedure, place, or time where a physician is operating and indicates the correct behavior as the basis of any clinical practice when applied to medicine [3]. The genesis of these principles lies in 400 B.C. in ancient Greece, with the Hippocrates of Kos; this oath requires physicians to treat patients according to their ability and knowledge, exercise their judgment, maintain a relationship based on confidentiality, and do no harm whilst benefitting the patient [4]. This oath laid the foundation of Western medical deontology (from the Greek *deonloghìa*[i.e., the study of duty]) [3, 4].

These principles have been adapted over the centuries following the evolution of medicine and society until the concept of “four principles plus scope” was established in the 1980s. According to these principles, ethical issues in healthcare are based on four commitments: (1) respect for patient autonomy; (2) Hippocratic concepts of beneficence and non-maleficence; (3) justice (i.e., equality); and (4) concern for scope of application. In AM, the scope of application is to achieve a more pleasant look, which may be desired by a patient for several reasons (e.g., solving a marriage crisis or making it easier to find a job), requiring an intimate understanding of a patient’s psyche, from the ancient Greek *psykhé*(i.e., soul) [4]. The concept of autonomy recognizes the capacity for self-determination using agency (the awareness of oneself as having desires and intentions and acting upon them), independence (the absence of any influences that control what a person does), and rationality of pondered decision-making [5]. The capability of a patient to be independent in their therapeutic choices is strongly linked to the Hippocratic concept of confidentiality, from the Latin *cum fides*(i.e., with trust), which requires those who possess privileged information not to share it without the permission of the patient or the confider, and respecting their autonomy and right to privacy [6].

The world where a physician operates today is not the same as that for Hippocrates, nor is it the same as the 1980s. AM specialists now rely on techniques that did not exist twenty years ago, and the number of procedures performed is soaring along with cost reductions, making it more affordable. The ongoing evolution in AM has not occurred alongside a clear definition of who is entitled to perform AM procedures, the location of where these procedures are performed, and the guidelines that should be followed. This development has generated misunderstandings, even among physicians of other specialties, who often regard AM as a chance to expand their patient base to round out their earnings (as if any health-related degree, sometimes not even that, and weekend training would allow anyone to practice in AM) [2].

The economic aspect of the practitioner's profit is legitimate since AM improves patient quality of life (QoL) but does not have the prerogative of saving lives; however, this business connotation has often relegated AM to the rank of a frivolous and commercial subspecialty [2].

AM also treads the constantly changing terrain of embellishing and rejuvenating, also known as “cosmetics,” from the ancient Greek *kosmetikòs* (i.e., adorned). This terrain is influenced by beauty standards, which were dictated in the ancient world by the arts (first and foremost paintings and sculptures), by rulers (e.g., from the Pharaohs to Marie Antoinette), by movie and pop music stars until the end of the last millennium, and today by the most powerful media ever conceived, namely the World Wide Web. Once again, word etymology reveals something unexpected: “beauty” comes from the Latin *bellus*, a diminutive of the adjective *bonum* (i.e., good). This concept was already present in ancient Greece, with the expression *kalòs kai agathòs*(i.e., beautiful, and good), emphasizing the association between moral and physical qualities to indicate perfection. This idea leads to a reinterpretation of beauty from purely aesthetic to the addition of ethical connotations of wholesomeness and virtuousness therefore AM implies achieving a result that is not only aesthetically pleasing to the senses but is also healthy [7].

In whatever way beauty is defined, it is ephemeral and was described by Ovid as *forma bonum fragile est* (i.e., beauty is fragile), which translates into the struggle to maintain it by resorting to AM and deceive time and nature (an apparent contradiction since physicians in ancient Greece studied the *physis* (i.e., nature) by going along with it rather than against it.

The purpose of this article is to analyze the relationship between AM and ethics, focusing on the role of social media, safety and responsibility issues, complexity of the doctor−patient relationship, search for harmonization, recognition of the AM physician within professional peer groups, and aims at the defining patient-centered ethics in AM, with the awareness that AM, despite its uniqueness, has always been an integral part of medicine.

## Discussion

### AM: past and present

Treatments aimed at modifying the human body have been known for centuries. Ancient Egyptians carried out skull elongation, reconstructions of the nose, ears and mouth were performed in the 6^th^ century B.C. in India, and descriptions of drooping eyelids and gynecomastia procedures are present in the Turkish literature of the 11^th^century [8]. In the 19^th^century, facelifts were carried out with a solution of arsenic and lead, and the invention of the syringe allowed the injection of substances beneath the skin for aesthetic purposes, using a series of not-ideal injectables, such as paraffin [9]. The 20^th^century saw an increase in the popularity of invasive surgery, made possible by new anesthetic agents, analgesics and antibiotics, which enabled the face reconstruction of World War I disfigured soldiers [10]. Jacek Maliniak, a surgeon, opened the first plastic surgery clinic in the United States (US) in 1921 [8]. A few Hollywood actresses had their ribs removed to achieve the slim “wasp” waist, even Marilyn Monroe reportedly had a sponge chin implant to make her face shapelier [11], and the 60s were the golden age of breast implants since the first breast enlargement surgery using silicone implants was performed in 1962 [11]. The new millennium saw the expansion of botulinum toxin for smoothing out wrinkles, more biocompatible rejuvenating and regenerative injectables such as hyaluronic acid (HA) and platelet-rich plasma for hair restoration, and new techniques involving the use of adipose-derived stem cells, which are giving promising results in the field of regenerative medicine and AM [12–16].

According to the International Society of Aesthetic Plastic Surgery worldwide 2020 data, there was a decrease of 1.8% for all AM procedures, essentially due to safety and financial concerns during the coronavirus disease 2019 (COVID-19 pandemic) [17]. In addition, plastic surgery procedures for aesthetic purposes decreased by 10.9%, while nonsurgical procedures continued to increase, but this increase was less than previously observed [17]. The most common surgical procedures worldwide remained as breast augmentation (16% of all procedures), liposuction (15.1%), eyelid surgery (12.1%), rhinoplasty (8.4%), and abdominoplasty (7.6%) [17]. The top five nonsurgical procedures also remained consistent: botulinum toxin (43.2% of total), HA (28.1%), hair removal (12.8%), nonsurgical fat reduction (3.9%) and photo rejuvenation (3.6%). Interestingly, despite the overall reduction in surgeries, rhinoplasty and brow lift surgeries, as well as nonsurgical facial rejuvenation continued to increase in 2020 but decreased in both 2018 and 2019 [17]. Observing the age distribution for all procedures, the highest proportion of rhinoplasty procedures were for 19−34-year-olds (67.9%), while 35−50-year-olds accounted for most botulinum toxin procedures (50.2%) [17]. Dermal fillers have potential therapeutic applications in children with atrophic disorders such as lipoatrophy and morphea but safety and efficacy studies in the pediatric age group are limited and a multidisciplinary assessment is recommended [17]. A smaller but steadily increasing percentage of AM patients are men; the American Society of Plastic surgery reported a 99% rise in cosmetic procedures performed on male patients between 2001 and 2021 [17]. Men first seeking aesthetic procedures non-invasive (i.e., botulinum toxin injections, HA fillers) and hair removal, followed by eyelid surgery and liposuction [17]. By geographical distribution, the US consolidated its position as the number one country where surgical procedures were performed (14.7%), followed by Brazil, Germany, Japan, Turkey, Mexico, Argentina, Italy, Russia, India, Spain, Greece, Colombia, and Thailand [17]. Hospitals continued to be the primary facility for surgical procedures (43.8% worldwide), apart from the US, where office facilities and free-standing surgical centers were more commonly used [17].

By its nature, AM does not include life-saving interventions, therefore a rhinoplasty undoubtedly does not pose ethical dilemmas like the case of an advanced pregnancy termination for an obstetrician or gynecologist, or the treatment of a patient with advanced cancer for an oncologist. In 2020, 10,129,528 surgical and 14,400,347 nonsurgical procedures were reported worldwide; as the number of AM procedures is rising, several issues surrounding AM practice need to be explored because the patient audience is changing, leading to pressure and expectations on the AM physician in the absence of globally-recognized ethical guidelines [17]. Because the physician has traditionally been identified as a healer, the main question arising is whether AM is solely a business or intended to benefit patients as an integral part of the healthcare system [18].

### The World Wide Web: a monster or an opportunity?

Whereas the aesthetic standards of the past represented by the athletic statues of the Hellenic Kuroi, Raphael's delicate depiction of Madonna, or Renaissance paintings of voluptuous women were the result of rigorous studies of body proportions, today, the World Wide Web proposes a beauty standard that often clashes with realistic anatomical principles. Beauty standards are currently heavily set by social media, and developed countries have spread their prototypes of beauty globally, influencing the original concepts held by other cultures and ethnic groups, leading to an upsetting uniformity [19].

While Facebook remains the most heavily used platform by 68% of adults in the US [20], there has been rapid growth in the use of the photo-sharing application (Instagram), rising from a usage rate of 28% in 2016 to 35% in 2018 [20]. Originally meant to make communication among people faster, now, social media platforms have integrated functionalities to modify images, and the most used filters are those that rejuvenate appearance by making the skin smoother, the complexion brighter, the eyes lighter, and the figure slimmer. In 2017, the American Academy of Facial Plastic and Reconstructive Surgery recorded that 55% of surgeons reported seeing patients seeking surgery to look better in selfies (a 13% increase from the previous year) [20]. More interestingly, above-the-shoulder surgical procedures compared with below-the-shoulder ones significantly increased following February 2020, likely due to the spread of video conferencing that fuels the need to look better on the screen, which can be considered more important than looking better in real life (the “Zoom-effect”) [21].

Characteristics of the typically AM candidate have shifted from well-resourced female patients to a patient with varying social, ethnic, and cultural backgrounds, who are also often younger and better-informed. Because the model presented by social media is perpetually young, the increase in rejuvenation procedures is requested at an increasingly early age [17], creating the paradox of a society that is long-lived but does not want to show it [22].

On the other hand, social media is also a tool for AM specialists to promote their practice and services and communicate directly with their potential patients through live broadcasts of procedures, raising ethical concerns about whether this is merely entertaining rather than a genuine representation of care [23]. However, social media also has the potential to foster in patients the pursuit of achievable aesthetic goals, raising awareness that AM can be a tool that improves QoL and is not just a temporary palliative. The letter “C” in “ETHICS” should stand for care because AM is, first and foremost, a branch of medicine; therefore, its mission is to care and be beneficial, which implies accompanying the patient along the entire therapeutic pathway, from the first consultation to post-procedure follow-up.

### Physician−patient alchemy

The major determinant for a successful cosmetic procedure is a healthy authentic physician-patient relationship, which starts with appropriate patient selection. It is important that physicians always bear in mind conducting their practice ethically, for the right reason, and on the right person, and patient personality profiling is of extreme importance. Patients who have previously been to numerous clinicians reporting suboptimal outcomes may suffer from body dysmorphic disorder, and performing a procedure on such individuals is almost guaranteed to be followed by dissatisfaction. In addition, very detail-oriented patients may show obsessive-compulsive disorder traits [24]. The AM specialist can refuse to perform a procedure in the presence of a personality disorder rather than feed a desire that is a symptom of a mental health condition [25].

Patients referring to AM specialists often want to change their aesthetic appearance but are also heavily concerned with how they will look after treatment. For this reason, the principle of “cosmetic conservationism” should always be well explained, and it should be noted that the goal of AM procedures is to make patients look better but still like themselves afterwards. With all the options of changing our appearance, it is theoretically possible to change your appearance so much that those who don’t know you well wouldn’t be able to recognize you, and this must be avoided [24]. A PPR based on beneficence-in-trust also implies honesty on the part of the physician to explain that the procedure outcome may differ from the patient's initial request concerning the starting situation and goal [26]. The letter “T” in “ETHICS” should stand for trust because the relationship between physician and patient must be based on mutual trust and a common language whereby the patient clearly expresses their expectations, and the physician exercises their clinical judgment to accommodate, downgrade, upgrade, or reject the procedure.

A recent global survey was carried out among patients and physicians within AM to assess disconnections related to the PPR. Results revealed several discrepancies regarding the ideal age of initiating treatment, treatment goals, concerns over the anatomical treatment areas, and barriers to seeking AM procedures. These differences underline that the physician and patient must find a common and effective communication ground to avoid disappointment and establish limits between what is desired and what can be realistically achieved [27].

A substantial generational difference is observed for the AM patient population. “Baby Boomers (born between 1946 and 1964)” who are usually wealthier, less frugal, and more willing to achieve long-lasting results, while on the other hand, younger individuals belonging to the Millennial (born between 1981 and 1994/6) generation are more likely to “shop around” and try one treatment first to see if they like the results, and then undergo other procedures. The AM specialist must know these differences to establish proper PPRs and recommend the appropriate treatment for individual needs and economic means.

### The quest for harmony

Harmony comes from the ancient Greek *armonìa* (i.e., connection); in AM, creating a harmonious face or body implies new forms and geometries that coexist with the existing ones without clashing. In our view, the letter “H” in “ETHICS” should stand for harmony because besides any cultural or evolution-driven anthropometrical standards, the pursuit of beauty cannot disregard the anatomical features of individual patients and their specific interconnections, with a balanced aesthetic vision of the individual.

The Golden Ratio (i.e., 1.618) represents the mathematical proportions the human eye finds most pleasing. Interestingly, these dimensions are found everywhere in nature and have been applied to art, while many studies have attempted to prove that the secret of human beauty relies on them [28–30]. However, attempts to prove that the degree of a face’s beauty is due to applying a mathematical formula have led to inconclusive results [31]. In addition, a patient's anatomical features pose constraints on applying the Golden Ratio: if mathematical and anthropometric principles are strictly followed, the patient would have to undergo multiple procedures to reach overall geometrical “perfection”. On the contrary, respect for beauty is, first and foremost, respect for biology and the unique proportions of a face, resulting from individual millennia-long genetic history, rather than algebraic proportions [31].

In this respect, AM fits equally well with other clinical specialties and with the wider concept of precision medicine, which considers the characteristics of the individual as a response to the relationship between the environment, patient lifestyle, genetic factors, and therapeutic choices [32].

If a patient wants to maintain a youthful appearance, the AM physician is expected to reduce wrinkles and laxities and while considering how the individual’s appearance will vary with age. The relationship between the AM physician and the patient becomes a confidential partnership that does not end but instead involves ongoing follow-up. The desire to preserve a long-lasting, youthful physical appearance involves repeated procedures to turn the inevitable change over time into something pleasant and authentic (derived from the Greek *aùtentikòs,* i.e., author).

AM can now rely on technologies that are increasingly more efficient, less invasive, and respectful of the natural physiognomy of a face, as in the case of fillers based on HA (a molecule that is a natural component of skin dermis). Depending on the formulation, treatment area, and injection plan, HA fillers allow different goals (ranging from attenuating superficial and deep wrinkles to bioremodeling) to be achieved. HA fillers are used for wrinkle prevention in younger patients and for conservative and regenerative action in the older age groups thanks to their moisturizing, elasticizing, and antioxidant properties, which are consolidated over the course of treatment and demonstrated by a growing number of clinical studies. It is no coincidence that HA fillers are currently amongst the most popular procedures, since they are seen as a malleable and beautifying intervention with a regenerative and healing aspect, which is able to restore beauty along with tissue function, i.e., not just for vanity purposes, but also for maintaining health [15, 16, 33].

Moreover, in AM, the concept of prevention of ageing applies; harmony with outcomes after treatment is achievable, but not in all cases equally since baseline will vary according to genetics, age, and lifestyle.

### Safety and responsibility

“Safety” comes from the Latin *securus* (i.e., without worry), which, when applied to the medical field, means that the patient, procedure, place where it is performed, and the person who performs it must be hazard-free. “Responsibility” comes from *responsa* (i.e., response), meaning that the physician is held accountable and must answer for their actions; however, another fascinating hypothesis traces the origin of the word back to the Latin expression *res-rem ponderare*, which means to assess all the involved factors in a situation before acting. In our view, the letter “E” in “ETHICS” should stand for education because the AM physicians cannot improvise; their professional path cannot be separated from ongoing education and constructive exchange with peer groups to ensure the patient is “in the best possible hands.”

AM procedures performed by physicians with no formal training are an everlasting, critical issue, especially now since the rising popularity of non-invasive procedures has led to an increasing number of non-physicians (e.g., cosmetologists, aestheticians, and electrologists) providing these services without appropriate medical education or formal training in cutaneous medicine, cosmetic surgery procedures, clinical aspects of related techniques, and patient engagement strategies for post-treatment follow-up care [31]. This issue, along with the variability of regulations among countries, has created a dangerous blur between the lines of what constitutes a medical procedure versus a beauty treatment and has led to the emergence of hybrid medical spas and retail clinics, which rarely discuss safety hazards sufficiently and promise access to the latest medical technology at affordable prices, miraculous results without side effects, and a quick recovery time [34]. On the other hand, office-based and outpatient surgery procedures have increased, thanks to improvements in surgical techniques and the presence of safer anesthesia and more effective analgesics. Despite their advantages, office-based surgery can represent safety hazards because an AM physician’s certification, equipment, surgical procedures, and emergency backup are currently not subject to the same regulations and inspections as that within a hospital setting [35]. In our view, the letter “S” in “ETHICS” should stand for safety because the practice of AM cannot be separated from the minimization of all risks before, during, and after the procedure; this is based on the competence of those who perform it, the safety of the place where it is practiced, and a careful patient history assessment.

Informed consent should be sought after the physician sets realistic expectations by carefully explaining the procedure and any related risks. Moreover, to minimize adverse events during or after treatment, it is an intrinsic part of professional ethics to meticulously collect a patient’s medical history, including any comorbidities and allergies, as these could make the procedure more risky, unfeasible, or feasible but with suboptimal outcomes [36]. If the patient has contraindications, the ethical principle of autonomy states that the patient can decide to undergo the procedure anyway; however, the AM physician has the right to refuse to perform it if it is not ameliorating or beneficial. A statement of ethical principles developed by the World Medical Association emphasizes that “The patient cannot demand health treatment contrary to the law, professional ethics, or good clinical practices; in the face of such demands, the physician has no professional obligations” [37].

Seven criteria are defined for informed consent (competence to understand and decide; voluntary decision-making; disclosure of material information; recommendation of a plan; comprehension of terms and decision in favor of a plan; and authorization of the plan) [38]. Only when all these criteria are met can the informed consent be given. Critically, when the patient is an adolescent, informed consent must be given by the parent or legal guardian. An ethical and deontological dilemma occurs when the parent/guardian has the responsibility to provide/refuse consent for an adolescent's desire for cosmetic improvement, which can sometimes be an intimate need that is not easy to understand except by the individual requesting this change [39]. Therefore, the AM physician’s role is paramount to establish a relationship with the patient and those with legal responsibility for the patient to reach a beneficial and satisfying decision for all stakeholders involved [39].

### Class action

Because AM is often misunderstood, even within the academic world, it is an ethical decision by the AM physician to practice it skillfully, consistently, and thoughtfully. For the AM specialist to stand out from those who practice it without any real qualifications or titles and become recognized as a medical professional in all regards, it is necessary to achieve the status of a fully-fledged expert through ongoing training and acquired experience. Moreover, the professional exchange of information with colleagues globally broadens the cosmetic perspective by relating to professionals from different countries who face patients who desire different aesthetic outcomes [24]. Discussing clinical cases can help improve techniques and share common deontological issues related to the PPR, management of expectations, and limits to be respected (the most ethical path to pursue).

Collaboration and knowledge exchange between peers also allow for more in-depth research in AM with which pharmaceutical companies and patients are involved. Scientific investigation in AM increasingly has an ethical focus that provides aesthetic enhancement associated with improved tissue quality that is stable over time. Because AM crosses multiple specialties, from plastic surgery and maxillofacial surgery to dermatology, it is necessary to act now to create a sense of identity and belonging that can only be based on a shared ethical intent that views the patient at the center. In our view, the letter “I” in “ETHICS” should stand for identity because in a society based on rapid standardization of aesthetic standards, it is important for the AM physician to carry out procedures while preserving a patient’s uniqueness and authenticity over time.

## Conclusions

In a society increasingly based on unremitting visual stimuli, there has been an exponential growth in the number of AM procedures. This trend raises questions about maintaining professional, ethical principles and medical deontology with the growing popularity and commercialization of AM treatments. Since the perception of our body image (i.e., our aesthetics) impacts our mental health, this leads to issues about the role, extent, and ethical principles of medical specialties that focus solely on attaining “beauty.” According to the World Health Organization (WHO), health is not only defined as the absence of any disease or disability but more widely and holistically as “a state of complete physical, mental and social well-being” [40]. Therefore, it is evident that aesthetic medicine is a medical discipline and must be closely associated with ethically pertinent conduct.

## Data Availability

Not applicable.

## Abbreviations

AM: aesthetic medicine
COVID-19: coronavirus disease 2019
HA: hyaluronic acid
PPR: physician-patient relationship
QoL: quality of life
US: United States
